# An update on genetic frontotemporal dementia

**DOI:** 10.1007/s00415-019-09363-4

**Published:** 2019-05-22

**Authors:** Caroline V. Greaves, Jonathan D. Rohrer

**Affiliations:** Department of Neurodegenerative Disease, Dementia Research Centre, UCL Queen Square Institute of Neurology, Queen Square, London, WC1N 3BG UK

**Keywords:** Frontotemporal dementia, Neurogenetics, Tau, Progranulin, C9orf72, Biomarkers

## Abstract

Frontotemporal dementia (FTD) is a highly heritable group of neurodegenerative disorders, with around 30% of patients having a strong family history. The majority of that heritability is accounted for by autosomal dominant mutations in the chromosome 9 open reading frame 72 (*C9orf72*), progranulin (*GRN*), and microtubule-associated protein tau (*MAPT*) genes, with mutations more rarely seen in a number of other genes. This review will discuss the recent updates in the field of genetic FTD. Age at symptom onset in genetic FTD is variable with recently identified genetic modifiers including *TMEM106B* (in *GRN* carriers particularly) and a polymorphism at a locus containing two overlapping genes *LOC101929163* and *C6orf10* (in *C9orf72* carriers). Behavioural variant FTD (bvFTD) is the most common diagnosis in each of the genetic groups, although in *C9orf72* carriers amyotrophic lateral sclerosis either alone, or with bvFTD, is also common. An atypical neuropsychiatric presentation is also seen in *C9orf72* carriers and family members of carriers are at greater risk of psychiatric disorders including schizophrenia and autistic spectrum disorders. Large natural history studies of presymptomatic genetic FTD are now underway both in Europe/Canada (GENFI—the Genetic FTD Initiative) and in the US (ARTFL/LEFFTDS study), collaborating together under the banner of the FTD Prevention Initiative (FPI). These studies are taking forward the validation of cognitive, imaging and fluid biomarkers that aim to robustly measure disease onset, staging and progression in genetic FTD. Grey matter changes on MRI and hypometabolism on FDG-PET are seen at least 10 years before symptom onset with white matter abnormalities seen earlier, but the pattern and exact timing of changes differ between different genetic groups. In contrast, tau PET has yet to show promise in genetic FTD. Three key fluid biomarkers have been identified so far that are likely to be helpful in clinical trials—CSF or blood neurofilament light chain levels (in all groups), CSF or blood progranulin levels (in *GRN* carriers) and CSF poly(GP) dipeptide repeat protein levels (in *C9orf72* carriers). Increased knowledge about genetic FTD has led to more clinical presymptomatic genetic testing but this has not yet been mirrored in the development of either an accepted FTD-specific testing protocol or provision of appropriate psychological support mechanisms for those living through the at-risk phase. This will become even more relevant as disease-modifying therapy trials start in each of the genetic groups over the next few years.

## Introduction

Frontotemporal dementia (FTD) is a heterogeneous neurodegenerative disorder presenting with distinct changes in behaviour, language and motor function. Despite often being considered as a rare disease, FTD is probably the most common form of dementia experienced in people under the age of 60, with an estimated lifetime risk of 1 in 742 [1]. The behavioural variant (bvFTD) is characterised by changes in personality, while the language variant (known as primary progressive aphasia, PPA) is typically associated with progressive speech production or comprehension difficulties [2, 3]. People with FTD can also develop motor deficits, either amyotrophic lateral sclerosis (FTD-ALS) or Parkinsonism, in the latter case often with specific features of a corticobasal syndrome (CBS) or progressive supranuclear palsy (PSP) [4–6].

## Heritability, genes and phenotype

### Heritability

**Fig. 1:**
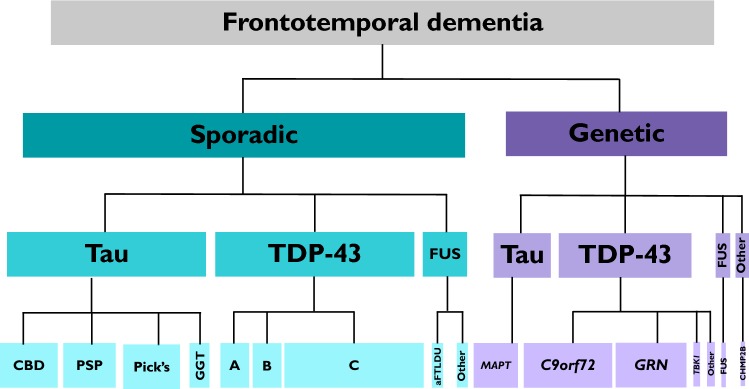
The landscape of the frontotemporal dementia spectrum disorders. About 70% is sporadic with approximately equal numbers of TDP-43 proteinopathies and tauopathies (including corticobasal degeneration, *CBD* progressive supranuclear palsy, *PSP* Pick’s disease, *GGT* globular glial tauopathy), and a smaller number of FUSopathies (including atypical frontotemporal lobar degeneration with ubiquitin inclusions, aFTLDU). About 30% is genetic with TDP-43 proteinopathies being the commonest cause (mutations in *C9orf72* (usually TDP-43 types A or B), *GRN* (type A), *TBK1* (types A or B)*, VCP* (type D), *SQSTM1,* and *TARDBP*) then tauopathies (mutations in *MAPT*), FUSopathies (mutations in *FUS*) and other proteinopathies (mutations in *CHMP2B*)

FTD is a highly heritable disorder but almost uniquely within the neurodegenerative disease spectrum, it is neither purely genetic (like Huntington’s disease, HD) nor a mainly sporadic condition (like Alzheimer’s disease) (Fig. 1). The extent of heritability of FTD has been the subject of a number of studies, with many of the initial investigations relying on the dichotomy between a ‘present’ or ‘absent’ family history. However, more nuanced family history scoring systems have been developed for FTD [7–9] revealing a complex picture of heritability. Using the modified Goldman score [7, 8] a strong family history [scores 1–3] was found in 31% [8], whilst using the Penn score, an equivalent strong family history [high or medium categories] was found in 26% [9]. All of these studies show variability in heritability across the clinical phenotypes, e.g. a strong family history has been found in 48% of people with bvFTD but only 12% of people with PPA [9]. Heritability of the motor phenotypes is less clear (mainly due to small numbers in most studies), e.g. a strong family history has varied from 10 to > 40% in FTD-ALS [8, 10, 11].

### Genes

The majority of the heritability of FTD is accounted for by autosomal dominant mutations in three genes: progranulin (*GRN*), microtubule-associated protein tau (*MAPT*) and chromosome 9 open reading frame 72 (*C9orf72*) [12, 13]. Each genetic group causes between ~ 5 and 10% of all FTD, with geographical variability in different case series (e.g. a predominance in Northern Italy and the Basque country of *GRN* mutations [14, 15]). Overall, *C9orf72* seems to be the most common worldwide cause of genetic FTD, followed by *GRN* and then *MAPT*. A list of pathogenic and other variants in these genes has been collated online in the AD&FTD Mutation Database (http://www.molgen.ua.ac.be/FTDmutations): 79 *GRN* and 45 *MAPT* pathogenic variants are currently described. However, a Pubmed search of mutations reported over the last 5 years in these genes identifies a further 35 *GRN* and 18 *MAPT* pathogenic variants not included in that database, i.e. 114 *GRN* and 63 *MAPT* mutations currently identified in total. This number excludes the majority of missense variants in *GRN*, many of which may be risk factors for Alzheimer’s disease rather than a Mendelian cause of FTD, although identifying pathogenicity is not always easy [16].

In recent years, mutations in an increasing number of genes have been associated with autosomal dominant FTD: *VCP* (2004), *CHMP2B* (2005), *TARDBP* (2008), *FUS* (2009), *SQSTM1* (2012), *CHCHD10* (2014), *TBK1* (2015), *OPTN* (2015), *CCNF* (2016), *TIA1* (2017). Cumulatively, they account for < 5% of all FTD, with most only found in a small number of families across the world. Recent studies have identified *TBK1* as probably the fourth most common genetic cause overall of FTD, accounting for between 1 and 2% of all cases (although the pathogenic nature of many of the reported missense variants remains unclear [17]). However, as with the major genetic groups, there is geographical variability: in a recent study of FTD in Sardinia, 8% of patients had a *TARDBP* mutation [18].

### Age at onset

Age at symptom onset is variable in each of the genetic forms of FTD, with intrafamilial variability (even within the same generation) of at least a decade in some families (particularly *GRN*). Whilst *MAPT* mutations are fully penetrant in most cases, both *GRN* [19] and *C9orf72* [20] mutations exhibit age-related penetrance with a small number of carriers in their 80s (and 90s) yet to develop symptoms. In both *GRN* and *C9orf72* carriers, *TMEM106B* has been identified as a genetic modifier, the association being stronger with *GRN* than with *C9orf72* [21]: a lower age at onset in *GRN* may well be related to carrying the risk allele, with homozygous carriers of the protective allele rarely found in symptomatic *GRN* carriers, suggesting that this may be a factor in age-related penetrance [22]. Another recently identified modifier of disease risk in *GRN* carriers, *GFRA2*, did not seem to affect age at onset [22]. However, a study of *C9orf72* carriers identified a locus on chromosome six containing two overlapping genes (*LOC101929163* and *C6orf10*) in which a polymorphism at rs9357140 was associated with age of onset: median age of onset in GG carriers was 6 years earlier than AA carriers [23]. The significance of the *C9orf72* repeat expansion length remains unclear, with no definitive evidence of an association with age of onset [24]. Little is known about factors that modify age at onset in the *MAPT* group, although a recent study suggested that ApoE ε4 carriers had a lower age at onset in tauopathies including *MAPT* mutations [25].

### Phenotype

The most common clinical presentation of all genetic forms is bvFTD, but all phenotypes within the FTD spectrum are observed. *MAPT* mutation carriers may have prominent semantic impairment but that is rarely a presenting feature, nor are other forms of PPA; however, CBS and, in rare cases, PSP may both occur, although never FTD-ALS. In contrast, *GRN* mutations can present as a PPA syndrome, either a nonfluent variant of PPA or a mixed phenotype, not clearly fitting into one of the three described subtypes [26]. CBS may occur either alone or in conjunction with PPA, but PSP and FTD-ALS are almost never seen. *C9orf72* expansion carriers may have an atypical neuropsychiatric presentation of bvFTD with associated hallucinations or delusions [27, 28], and significantly, family members of *C9orf72* carriers have a greater risk of psychiatric disorders including autistic spectrum disorders, psychotic illnesses including schizophrenia, mood disorders and suicide [27]. Unlike the other two major genetic groups, *C9orf72* expansions can cause FTD-ALS or ALS alone. PPA is a rare phenotype but is usually a nonfluent variant when present, and similarly parkinsonian disorders can occur but are infrequent as a presenting syndrome. Also unlike the other genetic groups, hyperkinetic movement disorders may occur, and *C9orf72* is said to be associated with a Huntington’s disease-like phenotype on some occasions [29].

The phenotype in the other genetic groups is less clear. *TBK1* mutations can cause bvFTD, PPA, CBS, FTD-ALS and ALS alone—this unique combination within a single family can be particularly suggestive of a *TBK1* mutation. *TBK1* and *TARDBP* mutations can both be associated with focal temporal lobe atrophy and a semantic variant PPA [18, 30, 31], an unusual genetic FTD phenotype as this variant of PPA is almost always sporadic.

## Natural history studies and biomarkers

Until recently, clinical studies of genetic FTD have been small and single centre. However, the Genetic FTD Initiative (GENFI) started recruiting in 2012 and now encompasses 25 centres across Europe and Canada (http://www.genfi.org.uk). This is a natural history study with detailed phenotyping of both presymptomatic and symptomatic mutation carriers [32]. In the US, a similar study (ARTFL/LEFFTDS) has been running for the last few years. Collaboration across natural history studies of genetic FTD across the world has started through the creation of the FTD Prevention Initiative (FPI: http://www.genfi.org.uk/fpi.html), aiming to share information and inform future clinical trial design.

**Fig. 2:**
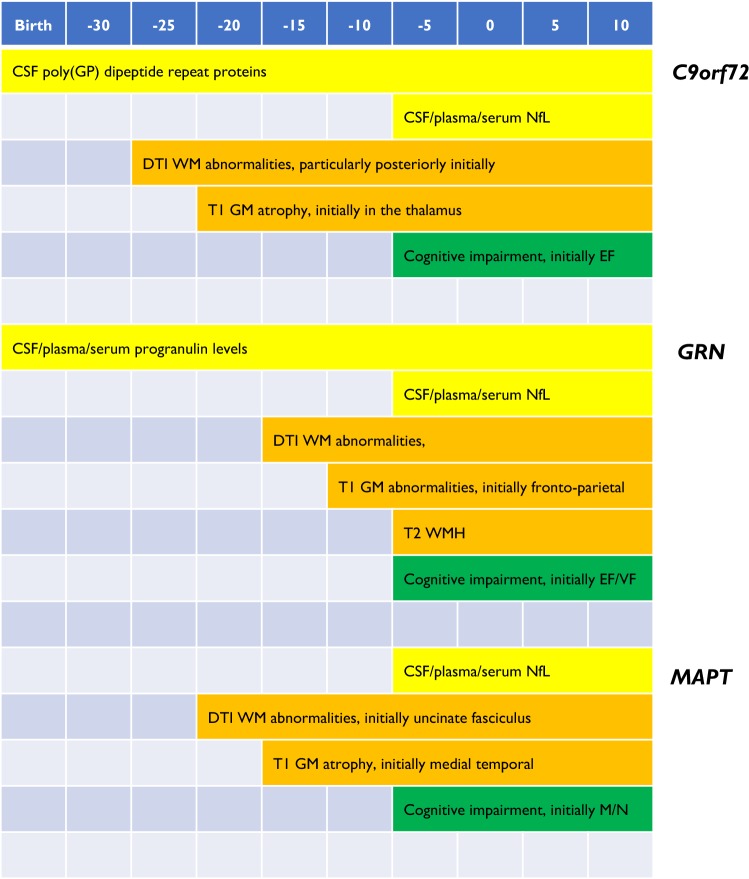
Schematic of fluid, imaging and cognitive biomarker profiles across the lifespan of *C9orf72*, *MAPT* and *GRN* mutation carriers. *NfL* neurofilament light chain, *DTI* diffusion tensor imaging, *WM* white matter, *WMH* white matter hyperintensities, *GM* grey matter, *EF* executive function, *VF* verbal fluency, *M* memory; *N* naming

Much of the work being performed in these studies (and in other single centre investigations) over the last few years has aimed to develop validated biomarkers that robustly measure disease onset, staging and progression (Fig. 2). The following sections highlight recent work in this field.

### Cognition

Neuropsychometric measures are abnormal in presymptomatic carriers around 5 years prior to expected symptom onset [32]. Whilst executive function deficits seem common across the different genetic groups, specific patterns of cognitive decline have been identified at a presymptomatic stage in *MAPT*, *GRN* and *C9orf72* carriers [32]. A number of studies have now shown that *MAPT* carriers have both naming and episodic memory difficulties presymptomatically [32–34], consistent with early medial temporal lobe atrophy [32]. As mentioned above, whilst most people develop bvFTD, some develop PPA, and one study has shown that longitudinal preclinical decline on phonology and letter fluency tasks was predictive of conversion to a nonfluent variant PPA phenotype in *GRN* carriers [33].

### Neuropsychiatric and functional measures

Validated measures of psychiatric symptoms or functional decline are limited in genetic FTD. The Neuropsychiatric Inventory (NPI) has been the most studied, although was not designed with FTD in mind, and does not include all relevant psychiatric symptoms that are seen in FTD [35]. The Cambridge Behavioural Inventory (CBI) has been used in the GENFI study and has shown changes in proximity to symptom onset [32], although as with many behavioural questionnaires there can be variability over time in FTD. More specific measures of particular symptoms such as loss of empathy (e.g. the Interpersonal Reactivity Index) or impaired self-monitoring (e.g. the Revised Self-Monitoring Scale) have not yet been well studied in genetic FTD. In terms of measuring disease severity and decline in function over time, an adaptation of the Clinical Dementia Rating scale for FTD (commonly called the FTLD-CDR) shows promise in genetic FTD [35], as does the FTD Rating Scale (FRS) [36], but more detailed studies of these and other novel measures are required.

### Imaging

Grey matter atrophy and hypometabolism both appear to occur at least 10 years before symptom onset in genetic FTD, whilst white matter tract abnormalities are seen earlier [37]. However, there is variability both in timing and location between the different genetic groups.

#### Grey matter atrophy (T1-weighted MRI)

In presymptomatic *MAPT* carriers, atrophy is present about 15 years prior to symptom onset in the anterior and medial temporal lobes, orbitofrontal lobe and insula [32, 38], whilst in *GRN* carriers, presymptomatic atrophy can be observed in frontal, parietal, and insular cortex as well as the striatum around 10 years prior to symptom onset [32, 38]. Symptomatic *GRN* carriers commonly have a very asymmetrical pattern of brain atrophy, and this asymmetry can be observed around 5 years prior to onset [32]. *C9orf72* mutation carriers appear to have earlier grey matter volume loss than the other two groups, before the age of 40 [39], and potentially more than 25 years prior to symptom onset [32]. This appears to be particularly focused on the posterior thalamus and its cortical connections [32, 38, 39].

Volumetric MRI studies of genetic FTD have particularly highlighted the importance of subcortical structures in the pathogenesis of FTD, and more recent work using novel postprocessing techniques has aimed to study the subregions within these structures, e.g. there are differential patterns of atrophy within hippocampal subregions in the different genetic groups: *MAPT* mutation carriers had involvement of CA1-4, *C9orf72* expansion carriers CA4, CA1 and the dentate gyrus, and *GRN* mutation carriers the presubiculum and subiculum [40].

There has been less focus on longitudinal investigation of grey matter atrophy; however, rates of atrophy vary between genetic groups with faster rates in *GRN* mutation carriers during the symptomatic period (allowing measurement over short time periods: [41]) compared with the other groups. Around the time of symptom onset, there seems to be a more gradual progression of atrophy in *MAPT* mutation carriers but a rapid change in volume loss in *GRN* carriers [42].

Few studies have investigated disease staging of genetic FTD. One novel machine-learning methodology combining subtyping and staging identified genetic FTD subtypes and their stages over time from structural T1-weighted imaging alone [43]. Interestingly, whilst *GRN* and *MAPT* mutation carriers appeared to fall mainly into a single group, there were two distinct patterns of disease progression for *C9orf72* expansion carriers—it remains unclear pathophysiologically what differs between these two groups.

#### White matter hyperintensities (T2-weighted MRI)

A number of studies have now shown that white matter hyperintensities (which are generally an unusual finding in FTD) are characteristic of *GRN* mutations [44, 45]. This is mainly in symptomatic mutation carriers (although for unclear reasons only a subset of patients), but there is also an association in presymptomatic mutation carriers with time from expected symptom onset [45]. Pathological studies of these white matter hyperintensities suggest that they are not vascular but are associated with prominent white matter microglial activation and microglial dystrophy [46].

#### Hypometabolism (FDG-PET)

Patterns of hypometabolism commonly mirror the pattern of grey matter atrophy in genetic FTD [47–51], with presymptomatic deficits also shown around 10 years prior to symptom onset.

#### Structural connectivity (DTI)

Changes in white matter integrity are commonly measured with diffusion tensor imaging (DTI), although newer techniques such as neurite orientation dispersion and density imaging (NODDI) have recently been developed. Studies in genetic FTD suggest that changes can be observed as far back as 30 years prior to symptom onset [52]. As with grey matter atrophy, there appear to be distinct patterns of early white matter involvement in the different groups: presymptomatic *MAPT* mutation carriers have alterations in the uncinate fasciculus and parahippocampal cingulum, while *GRN* mutation carriers show involvement of the anterior and posterior internal capsule [52]. Presymptomatic *C9orf72* expansion carriers have earlier white matter tract pathology, which occurs in posterior tracts such as the posterior thalamic radiation, the posterior corona radiata and the splenium of the corpus callosum [52, 53]. A single study of NODDI suggests that it may be more sensitive than DTI for detecting early white matter change in *C9orf72* expansion carriers [54].

#### Functional connectivity (resting-state fMRI)

There have been fewer investigations of functional connectivity but small studies implicate particularly the salience network and a medial pulvinar thalamus-seeded network in presymptomatic *C9orf72* expansion carriers [53], the default mode network in *MAPT* mutation carriers [55] and a frontoparietal network in *GRN* mutation carriers [56–58].

#### Tau PET

Studies of novel radioligands developed to bind tau protein have so far not proven to be particularly helpful in FTD, binding much more strongly to paired helical filament (PHF)-tau found mainly in Alzheimer’s disease than to other forms of tau found in the primary tauopathies. However, two particular *MAPT* mutations (V337M and R406W) are associated with PHF-tau and have shown strong binding with the AV1451 tracer [59–61]. Unfortunately, there is also off-target binding of this tracer, with binding seen in non-tau diseases such as in *C9orf72* expansions, where the major pathology is TDP-43 [62].

### Blood and CSF biomarkers

The fluid biomarker field in genetic FTD has yet to identify many robust measures, e.g. neither CSF nor blood assays of tau or TDP-43 are yet to yield FTD-specific markers. However, recent work has identified three markers which will play an important role in forthcoming trials: neurofilament light chain (NfL), progranulin and poly(GP) dipeptide repeat proteins (DPRs).

Increased NfL levels (both in CSF and blood) reflect axonal damage and appear to be a measure of disease intensity, and predict progression and survival in genetic FTD [63, 64]. Levels are highest in *C9orf72*-associated ALS and lowest in *MAPT* mutation carriers [64]. Longitudinal analysis of samples seems to suggest that levels change not long prior to symptom onset in genetic FTD, increasing by three- to fourfold during conversion [64]. Whilst an increase in NfL is not specific for FTD, and levels are increased in multiple neurological diseases, evidence from other diseases suggests that a decrease in levels could be a measure of successful disease modification in trials [65].

Low serum, plasma or CSF progranulin levels have almost perfect sensitivity and specificity for detecting pathogenic *GRN* mutations [66, 67]. Levels are low from the earliest time period of presymptomatic genetic FTD that they have been measured [during adulthood] and are relatively stable over time [67]. CSF and plasma levels are relatively poorly correlated (*r* = 0.54: 67], and little work has been done to investigate measures that affect the variability of progranulin levels. This future research is important as increasing progranulin levels back towards normal levels (and therefore theoretically restoring normal progranulin function) will be a key biomarker for disease-modifying trials in *GRN* carriers.

Increased poly(GP) levels have been identified in the CSF of *C9orf72* expansion carriers both presymptomatically and symptomatically [68–70]. One study found slightly lower levels in presymptomatic expansion carriers compared with symptomatic carriers [70] but that has not been seen consistently. More work needs to be performed to understand variability further, but like NfL, decreasing levels of CSF poly(GP) post-treatment may be suggestive of disease modification in future trials.

A particular focus of biomarker research in genetic FTD is the development of markers of neuroinflammation. CHIT1 and YKL-40 are microglial markers that appear to be raised in symptomatic genetic FTD [71] with little evidence for a change during the presymptomatic period so far. In a small study, CSF sTREM2 levels were raised in *GRN* mutation carriers but not the other genetic groups [72].

## Clinical practice (Fig. 3)

### Symptomatic genetic testing

Testing in symptomatic patients with dementia has changed in recent years. Next-generation sequencing (NGS) panels are now available to test multiple genes at the same time—these have identified mutations causative of FTD pathology not just in those with an FTD clinical syndrome [73]. Issues that remain to be solved in clinical genetic testing include: how to decide the pathogenicity of certain variants (of which more are now found because of NGS); the exact length at which *C9orf72* expansions become pathogenic (as intermediate length expansions are not clearly causative of disease [74]); and what to do when no mutation is found in a family with autosomal dominant FTD. In terms of this latter problem, many available NGS panels do not include the more recently discovered genes such as *TBK1* and we have identified mutations in these genes by exome sequencing in those with negative NGS and C9orf72 sequencing [30]. However there are still a small number of families with a strong family history of FTD without a known genetic mutation. We offer genetic testing in our clinic to all those with bvFTD, even in the absence of a family history, as mutations have been found in around 10% of apparently sporadic cases of FTD [75]. In the other FTD clinical phenotypes, where the risk is lower of a genetic cause, we offer testing on an individual basis, mainly in those with a strong family history, but the identification of a PPA syndrome not fitting criteria for one of the three described subtypes is a red flag for consideration of testing (with the expectation of potentially finding a *GRN* mutation) [26, 76].

**Fig. 3:**
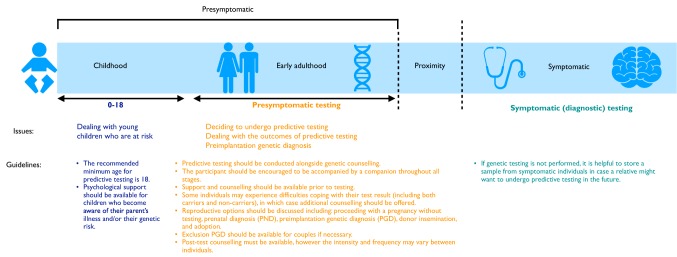
Genetic testing, counselling and support through the timecourse of genetic FTD. There is commonly a period in proximity to symptom onset of FTD where subtle symptoms may be present but diagnostic criteria have not yet been met—this requires careful assessment and discussion

### Presymptomatic genetic testing

Once a causal mutation has been established in a symptomatic relative, the option of predictive genetic testing can be raised with at-risk family members. While potential treatments for FTD are still lacking, appropriate clinical care for presymptomatic populations is integral. The genetic counselling and support systems in place lag far behind those seen in other neurodegenerative disorders. Whilst in practice the HD predictive genetic testing protocol is currently used as the gold standard [77], there are a number of key distinctions between HD and FTD which mean that the HD protocol may not be appropriate for the FTD population [78], including age-related penetrance, unpredictable age at onset of symptoms, and phenotypic heterogeneity. Similarly, access, experiences and attitudes towards predictive testing can vary depending on location [79], and future development of an FTD-specific protocol may be more suitable.

The HD predictive guidelines stress the importance of psychological evaluation in presymptomatic carriers, with others suggesting that psychological assessment is a necessary process for identifying an individual’s risk of experiencing an adverse psychological reaction to presymptomatic testing [80]. There remain a large proportion of individuals who live at-risk of FTD who decide against predictive testing—probably about 70–80% of this population [32]. These individuals receive little or no support as many will not have even been through genetic counselling, and little work has been done to identify their psychological needs. Initial research does suggest that rates of depression and mood disorders are higher even in non-carriers within FTD families [81]. One method of helping such individuals is the provision of specific support groups aimed at providing peer support and information about the at-risk period—the familial FTD support group in the UK is one such example (http://www.raredementiasupport.org/fftd/). Specific interventions at an appropriate time such as cognitive behaviour therapy or mindfulness have yet to be trialled.

## Clinical trials and emerging therapies

There are currently no disease-modifying therapies for genetic FTD but trials are now underway or planned in each of the three main genetic FTD groups. Antisense oligonucleotide therapy shows promise for both C9orf72 expansions [82] and *MAPT* mutations [83], whilst AAV gene therapy is a potential avenue for disease modification in *GRN* carriers [84, 85], although one study in a mouse model showed evidence of T cell-mediated toxicity [85]. Small molecule therapies and tau monoclonal antibodies are also being developed for tauopathies (with a potential for use in *MAPT* mutations) [86], and other options for *GRN* mutations include modification of proteins such as sortilin and HDAC that lead to increased *GRN* levels [87, 88].

## Summary

Much has been learnt about genetic FTD in the last decade, with the majority of autosomal dominant FTD now accounted for. The development of collaborative international multicentre natural history studies in GENFI and ARTFL/LEFFTDS has brought together researchers and families, and has helped to set the background for clinical trials that are now getting started and being planned. An associated support network for those living at-risk of genetic FTD is important and there is work to be done in improving this; but with the advent of specific gene-targeted therapeutics, there is hope in the community for the future outlook.
